# Reduced Lateral Mobility of Lipids and Proteins in Crowded Membranes

**DOI:** 10.1371/journal.pcbi.1003033

**Published:** 2013-04-11

**Authors:** Joseph E. Goose, Mark S. P. Sansom

**Affiliations:** Department of Biochemistry, University of Oxford, Oxford, United Kingdom; UNC Charlotte, United States of America

## Abstract

Coarse-grained molecular dynamics simulations of the *E. coli* outer membrane proteins FhuA, LamB, NanC, OmpA and OmpF in a POPE/POPG (3∶1) bilayer were performed to characterise the diffusive nature of each component of the membrane. At small observation times (<10 ns) particle vibrations dominate phospholipid diffusion elevating the calculated values from the longer time-scale bulk value (>50 ns) of 8.5×10^−7^ cm^2^ s^−1^. The phospholipid diffusion around each protein was found to vary based on distance from protein. An asymmetry in the diffusion of annular lipids in the inner and outer leaflets was observed and correlated with an asymmetry in charged residues in the vicinity of the inner and outer leaflet head-groups. Protein rotational and translational diffusion were also found to vary with observation time and were inversely correlated with the radius of gyration of the protein in the plane of the bilayer. As the concentration of protein within the bilayer was increased, the overall mobility of the membrane decreased reflected in reduced lipid diffusion coefficients for both lipid and protein components. The increase in protein concentration also resulted in a decrease in the anomalous diffusion exponent α of the lipid. Formation of extended clusters and networks of proteins led to compartmentalisation of lipids in extreme cases.

## Author Summary

Biological membranes are selective barriers which control the entry/exit of molecules to/from the interior of a cell. They are composed of a lipid bilayer in which are embedded many membrane proteins. Whilst the individual components of membranes are relatively well characterised, the lateral organization and dynamics of the membrane remain less well understood. The lateral mobility of constituent membrane species affects many processes, including how quickly proteins complexes form and protein recruitment occurs, how quickly lipids can be modified/lysed, and the formation of disordered and ordered microdomains. Biological membranes can contain as much as 50% protein. The dynamics of these crowded environments differ greatly from the sparsely populated membranes often studied *in silico* or *in vitro*. We use molecular dynamics computer simulations to quantify how mobility within the membrane decreases as the protein concentration increases. We calculate a baseline diffusion of both lipids and selected bacterial outer membrane proteins in the simplest of systems, namely a single protein in a large lipid bilayer patch. In this case diffusion can be correlated with the size of the protein. We observe how proteins affect the mobility of adjacent lipids. As the protein concentration within our systems increases we show that diffusion of both the proteins and lipids is reduced.

## Introduction

Lipid–protein interactions play an important role in the function and organisation of membrane proteins, either through macroscopic bilayer properties or via individual protein–lipid interactions [1]–[3]. For certain proteins, e.g. those involved with the regulation of membrane composition or maintaining an asymmetric leaflet distribution, the necessity of such interactions is evident, whilst for others that depend on lateral pressure or local bilayer deformation for function the interaction may be more subtle [4]. In order to understand the mode of action of these processes requires that we need to characterise not just the static structure of membranes but also their dynamic behaviour.

Cell membranes are crowded environments: the majority are composed of up to ca. 50% protein by mass corresponding to a membrane area fraction of ca. 25% or more occupied by proteins [5]. A similar degree of crowding may be found in membranes studied *in vitro* [6] or used in membrane protein based biosensors [7]. In addition to crowding *per se*, the spatial and compositional complexities of membranes may result in the formation of membrane protein clusters [8]. Much discussion as to the nature of cluster formation has centred around the formation of lipid rafts in certain membranes [9], but it should be noted that lateral interactions of crowded membrane proteins are a more general property of cell membranes [10] and are of importance in e.g. bacterial [11], [12] as well as mammalian cell membranes.

There has been considerable experimental and computational interest in crowding effects in cells in general (e.g. [13]–[15]). In particular there have been a number of computational and theoretical treatments of crowding in cell membrane environments (e.g. [4], [16]–[18]). Molecular dynamics simulations of crowded membrane systems have been relatively limited, in part due to their high computational demand, although such simulations of simple models of membrane proteins (e.g. [19]) have yielded valuable insights into peptide effects on lipid domain formation.

MD simulations have also been used to explore in detail the diffusion of membrane lipids, demonstrating the existence of correlated flows and motions within the bilayer [20]–[23] and of anomalous diffusion of lipids [24], [25]. More recently such studies have been extended to membranes including (single) protein molecules, revealing co-diffusion of protein and associated lipids, especially in membrane proteins such as the Kv channel which have a rather unique transmembrane architecture which leads to tight binding of a significant number of specific lipids [26]. This study takes a further step into understanding the dynamics of complex proteins and lipids in crowded membrane protein systems, and complements recent studies [27] focussing on anomalous diffusion. More generally, the current study should be seen in the context of a number of simulation studies exploring the influence of lipid bilayer thickness on membrane protein aggregation (e.g. [28]–[30]), and the effects of protein clustering on diffusive behaviour of lipids [27] and of membrane proteins [31]. The ‘in plane’ dynamic properties are likely to have important biological implications on higher level modelling of processes such as membrane protein sorting [32] and protein-induced membrane vesiculation [33].

In this study we have concentrated on a series of *E. coli* outer membrane proteins (OMPs): OmpA, NanC, FhuA, OmpF, and LamB. OMPs have a variety of functions especially for transport of solutes across the outer membrane (see Supporting Information Table S1) and offer a number of advantages as model systems which balance biological realism with relative simplicity. The OMPs all share a β-barrel architecture and so are unlikely to undergo any significant conformational change during the simulations. At the same time they are sufficiently diverse enough in size, oligomerisation state, and surface chemistry (see Supporting Information Table S1) to make a comparison worthwhile. In these simulations we employ a lipid bilayer composed of two lipids (POPE and POPG) representing the inner leaflet composition of the bacterial outer membrane [34]. Again this is a compromise between biological realism and simplicity. *In vivo* the outer leaflet is almost exclusively lipopolysacharride (LPS) which is a 5 to 6 tailed lipid and the inner leaflet is composed of POPE, POPG and cardiolipin. The bilayer we use here while not including LPS is perhaps more representative of the more common biological membranes containing a majority of two tailed lipids.

## Results

### Simulations

The simulations were designed to mimic the extent of protein crowding in bacterial outer membranes. Thus simulations were performed with between 1 and 16 OMPs in a bilayer of approximate dimensions 285×285 Å^2^ (corresponding to ca. 2500 lipids). This yields a protein density ranging from 1000 to 20,000 µm^−2^, corresponding to a fraction of the membrane area occupied by protein (θ) ranging from ca. 2% to ca. 50%. The upper level is comparable to the area fraction for OMPs in bacterial outer membranes [35], [36], in OMP-based biosensor membranes [7], and in recent high-speed AFM studies of OmpF-containing membranes [6]. The lower level is comparable to that employed in recent experimental studies of lateral diffusion of membrane proteins *in vitro* [37].

Five different OMPs were used, ranging in radius of gyration (*R_gyr_*) from 10 to 30 Å (see Supporting Information Table S1). For each protein, simulations were run in two bilayer environments (POPE and POPE/POPG) with 1, 4, 9 or 16 proteins in the bilayer patch (see Methods for details). Each simulation was run for at least 3 µs. This provides us with a substantial body of simulation data (a total of ca. 100 µs of simulation time) on which to base our analysis. However during the course of the analysis it became apparent that the dynamics of the two bilayer environments were identical and so only data from the mixed POPE/POPG bilayer is shown.

### Lipid Diffusion

Two dimensional lipid diffusion was initially studied in a lipid only POPE:POPG bilayer with no embedded proteins to characterise “bulk” properties. Fitting the lipid center of mass (COM) mean square displacement (MSD) versus time to equation 3 (see Methods) produces a straight line resulting in an exponent α = 0.99 indicating that diffusion is normal (Supporting Information Fig. S1). Subsequently fixing α equal to unity and re-fitting gives *D* = 8.5×10^−7^ cm^2^ s^−1^. There appears to be deviation from normal diffusion only for small *t* (<20 ns) where the MSD is elevated, this also corresponds to where the MSD of individual head groups diverges from the lipid COM.

**Figure 1:**
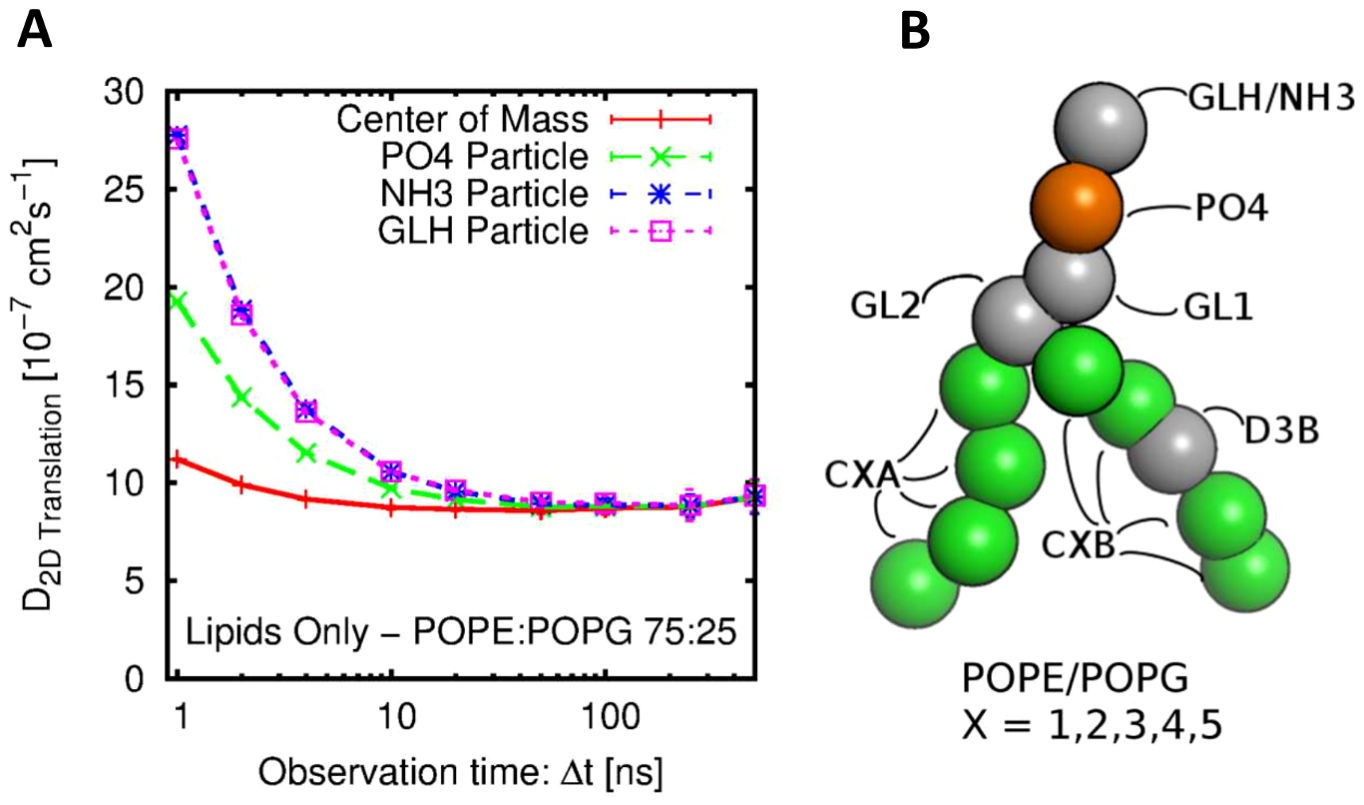
(A) Lipid diffusion coefficients as a function of observation time (Δt) for: lipid Center of Mass (red line), and for the phosphate (PO4, green lines) and choline (NH3, blue line) or glycerol (GLH pink line) particles of the headgroup. These diffusion coefficients were estimated from a 5 µ*s* simulation of a lipid bilayer containing a 3∶1 mixture of POPE/POPG. (B) Coarse-grained structure within POPE and POPG lipids illustrating the particle types.

By calculating the distribution of lipids from their initial position after observation time (*Δt*; Supporting Information Fig. S2 we calculate effective diffusion coefficients for different observation times, using equation 1 (see Methods), to categorise this more effectively. As expected the two-dimensional lipid diffusion coefficient is a function of the observation time (Fig. 1A) mirroring the result from above with large diffusion coefficients at low *Δt* converging to circa 8.5×10^−7^ cm^2^ s^−1^ at *Δt*>50 ns.

When individual head–group particles are used to track the lipid diffusion rather than the centre-of-mass (COM), the diffusion coefficients at small *Δt* (and MSD at small *t*) are elevated even further For smaller observation times, when compared to COM values, the increase in diffusion is exaggerated for PO4^−^ particles and further for NH3^+^ and GLH. The values for GLH (neutral) and NH3^+^ are identical indicating that particle electrostatics are not playing an important part in the mode of diffusion in the bulk. It further suggests that at low *Δt* we are sampling the particle vibrations (previously described [38], [39] as rattling in box) and that they are largely (but not completely) averaged out when considering the COM of the entire lipid, slightly less when considering the PO4^−^ particle (bonded at both ends) and the even less for the NH3^+^ and GLH particles (only bonded to one other particle, see Fig. 1B).

**Figure 2:**
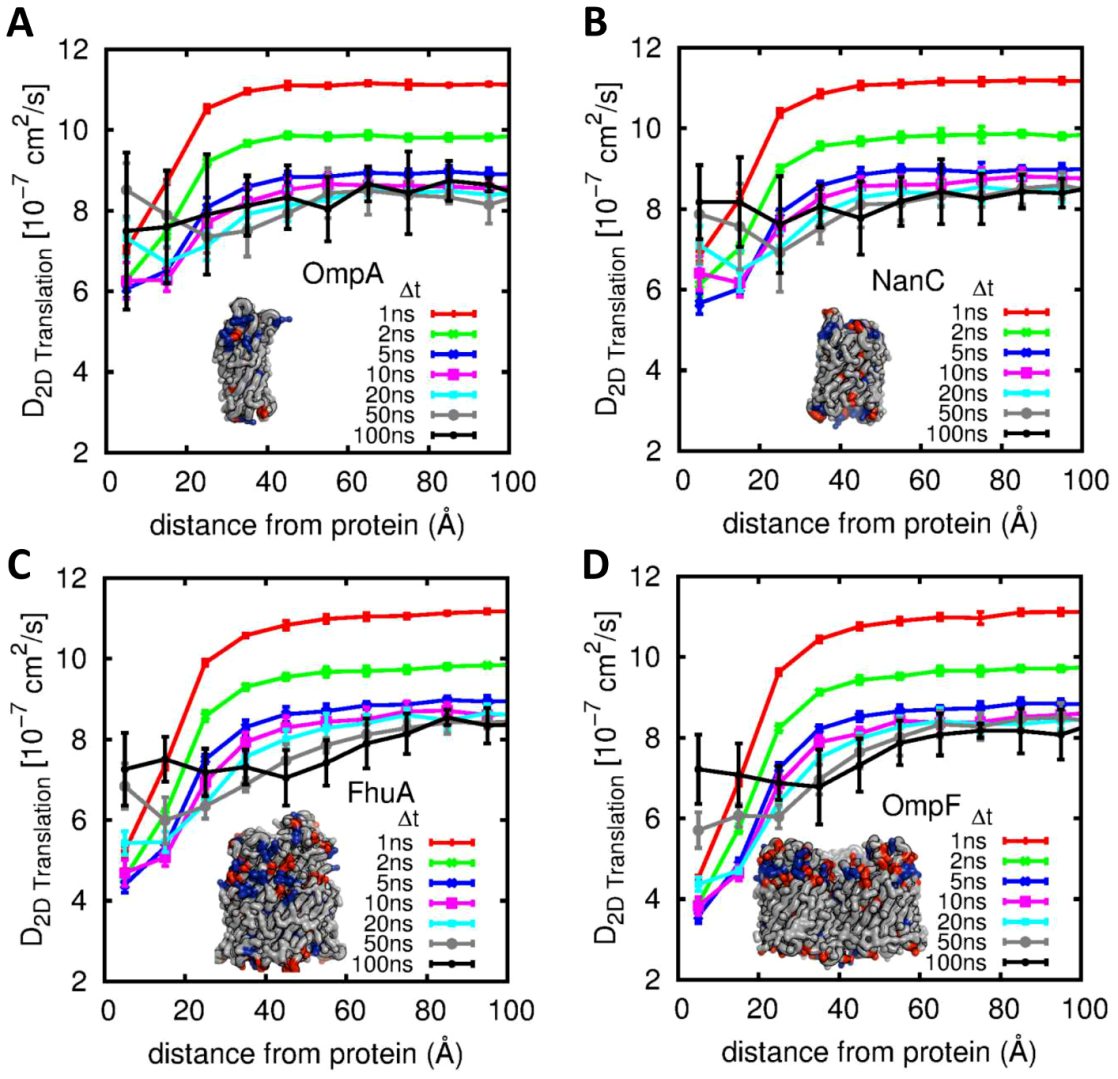
Phospholipid center of mass diffusion coefficients for the outer leaflet of the bilayer as a function of distance from protein and of observation time. Each point represents the diffusion of lipids within annuli of 10 Å width (i.e. a point at 5 Å represents lipids within the first annulus 0–10 Å from the protein surface). The data on each plot are calculated from 6 µ*s* trajectories of a single protein in a 3∶1 POPE:POGE bilayer. Error bars are the standard errors of 6×1µs sub-trajectories, Inset on each plot is the protein investigated showing acidic (red) and basic (blue) surface residues. The proteins and their PDB ids are: (A) OmpA (1BXW), (B) NanC (2WJQ), (C) FhuA (1BY3), and (D) OmpF (2OMF).

When we introduce an OMP into the system and calculate the COM diffusion of the outer leaflet phospholipids as a function of distance from the OMPs (Fig. 2), it is evident across all OMPs that diffusion close to the OMP is retarded in comparison to diffusion in the “bulk” (as has been seen for e.g. the Kv channel protein [26]). This retardation due to the proximity of the OMP is observed to penetrate as far as the 20–30 Å annulus from the protein surface, beyond which bulk diffusion is observed. The retardation is a little more marked around the larger trimeric proteins (OmpF ; with a value of ca. 40% that in the bulk at the surface of the protein) than it is around the smaller NanC and OmpA proteins (where the surface lipids diffuse ca. 60% as fast as the bulk lipid). Whether this is because of steric hindrance of the trimeric proteins presenting a less smooth surface with concave regions conducive to trapping phospholipids is not immediately clear. However, we do note that in previous studies of the Kv channel protein [26], which has an exceptionally infolded surface which results in tight binding of lipids [40], an even greater degree of retardation of lipid diffusion is seen.

When the same analysis is applied except using individual PO4^−^, NH3^+^ and GLH outer leaflet particles instead of the COM (Supporting Information Fig. S3 for NanC and OmpF systems) the retarded diffusion is again observed for all Δt, and is greater for GLH/NH3^+^ than for PO4^−^. For the close annular lipids around NanC the diffusion coefficients of the GLH/NH3^+^ particles (identical except charge) are identical whereas the GLH/NH3^+^ diffusion coefficients at small Δt around OmpF are not: the vibrations of the NH3^+^ particles are dampened by the large number of acidic residues in the vicinity. This is of interest, as it suggests a role for electrostatic interactions between protein and lipids in modulating the motion of the latter.

As the diffusion coefficients are characterised based on the initial lipid position relative to the protein if the observation time is much greater than the lipid residence time around the protein then lipids that start very close to the protein will also sample environments much further away. This is the reason that for long observation times the diffusion coefficients are not a function of distance from protein. The diffusion coefficients we calculate could be used in a model to predict the likely destination of a lipid from a given starting configuration.

### Leaflet Asymmetry

Membrane proteins are often asymmetric in terms of the distribution of charged residues between inner and outer leaflet ‘bands’ interacting with lipid headgroups (as reflected in e.g. the ‘positive inside rule’ for α-helical membrane proteins [41]). This is also the case for most OMPs, in part reflecting the asymmetric nature of the lipid composition of the outer membrane [34]. Thus in all of the OMPs in the current study, there are more charged sidechains in the outer than in the inner ‘interfacial band’ on the surface of the protein (see Fig. 2 insets and Supporting Information Table S1). If we measure the charge asymmetry from our simulations by calculating the number of charged side-chains that pass within 5 Å of each leaflet's (outer/inner) headgroup particles NanC has the smallest disparity (21/13) and OmpF has the greatest (72/18). We may exploit this difference between NanC and OmpF to explore the influence of electrostatic interactions on lipid dynamics.

**Figure 3:**
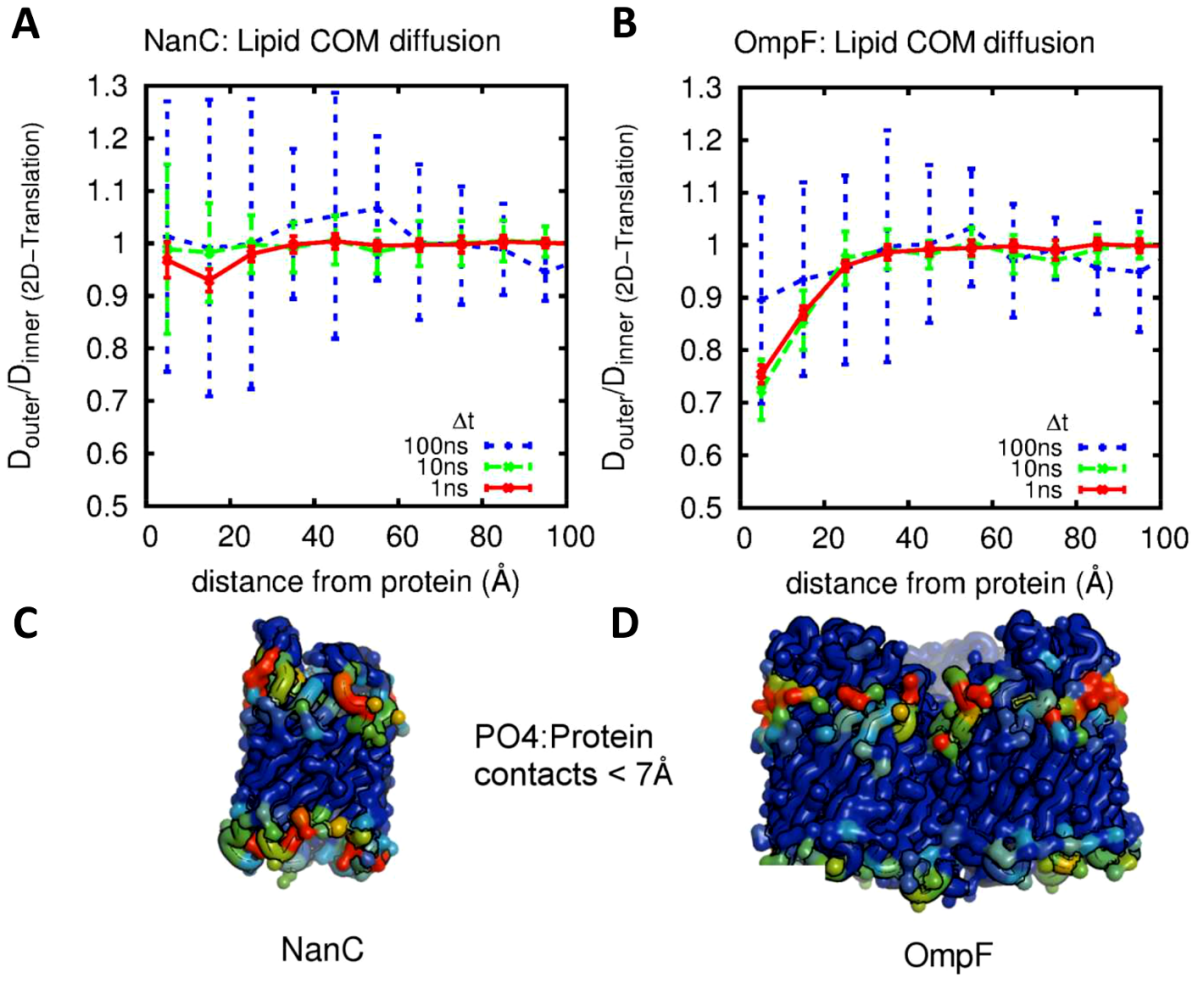
Leaflet asymmetry of diffusion coefficients illustrated for NanC and OmpF. Ratio of inner to outer leaflet center of mass diffusion coefficients for (A) NanC and (B) OmpF as a function of distance from protein at differing observation time. Error bars are the standard errors of 6×1 µs sub-trajectories, Asymmetry can be seen in the OmpF simulations for distances from the protein of <20 Å. (C,D) Cα trace representations of the corresponding proteins coloured on time averaged number of protein contacts (cutoff 7 Å) to lipid phosphate particles on a blue (0%) to red (100%) scale. Corresponding diagrams for all five proteins can be found in the supporting information, Fig. S4.

Comparing lipid diffusion coefficients between the inner and outer leaflets reveals that the mobility of annular phospholipids in the outer leaflet is generally less than that in the inner leaflet for all OMPs studied other than NanC (Fig. 3 and Supporting Information Fig. S4). The extent of asymmetry in diffusion between the leaflets can be estimated by examining the composition of the charged residues within each protein. The OMPs with fewer charged residues proximal to the lower leaflet headgroups than residues proximal to the upper leaflet headgroups exhibit a higher asymmetry. The extreme cases are NanC (little asymmetry) and OmpF (most asymmetry). NanC has a relatively high density of charged residues in the vicinity of the inner leaflet. (It is also more *rotationally* asymmetric than the other OMPs in terms of outwardly facing charged and aromatic residues.)

**Figure 4:**
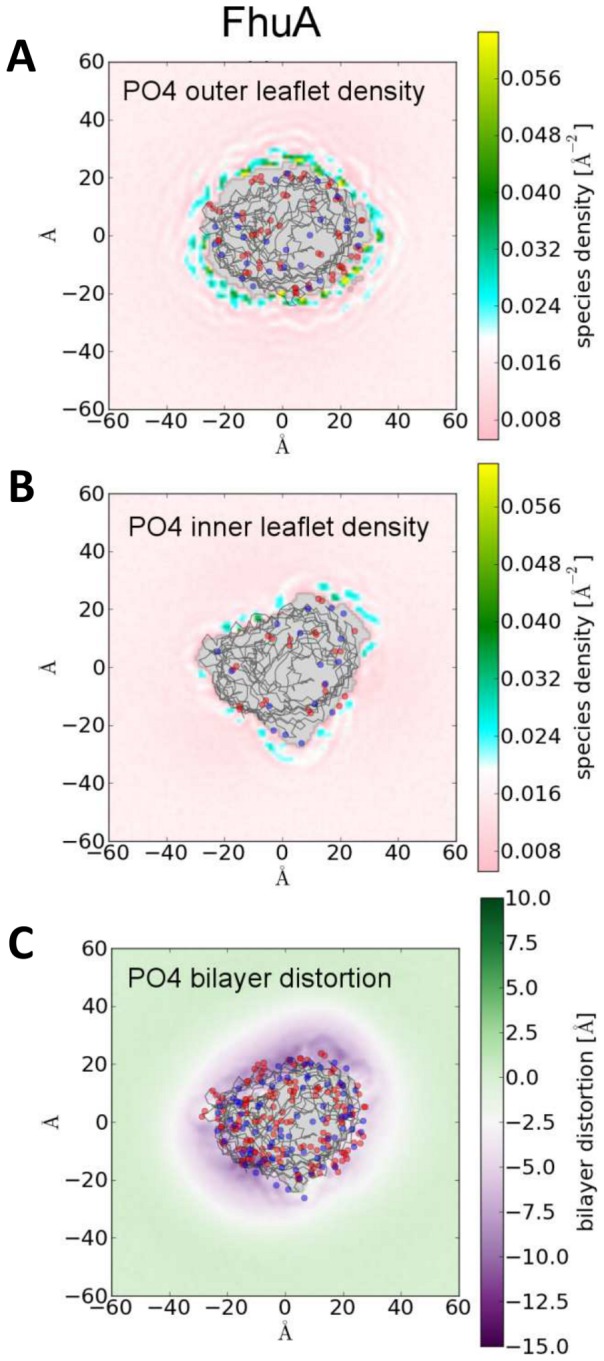
Time averaged two–dimensional phosphate particle densities (Å^−2^) around FhuA for the outer (A) and inner (B) leaflets. Proximal acidic/basic residues are shown as blue/red points. The Cα trace is shown in black. Corresponding diagrams for all five proteins can be found in the supporting information, Fig. S4. (C) Time averaged two–dimensional bilayer distortions from bulk thickness in the vicinity of FhuA. Bilayer thickness is calculated based on the minimum distance between the two closest PO4 particles in opposing leaflets. Acidic/basic residues are shown as blue/red points. The Cα trace is shown in black.

The asymmetry is directly reflected in the time averaged contacts made between the PO4 particles and the protein (Fig. 3 CD) and the time averaged lipid density around the OMPs (Fig. 4 and Supporting Information Fig. S5). For all OMPs the outer leaflet lipids exhibit a high density ring of PO4 headgroups directly around the protein sometimes extending out to a second ring (particularly FhuA; Fig. 4). The particularly high density regions (up to 5 times the bulk density) are roughly equally and densely spaced in the outer leaflet. In the inner leaflet whilst there is still an increase in density directly around the proteins there is a distinct lack of these regularly spaced high density regions (except perhaps in NanC; Supporting Information Fig. S5), and no evidence of any second radial density peak.

Thus, we can see that inner/outer leaflet asymmetry in the distribution of residues on the surface of the protein can in turn introduce dynamic asymmetry into the membrane as a whole. For the systems studied, such immobilisation is largely due to electrostatic interactions of protein and lipid headgroups. The exact location of the interaction density hotspots also gives us an insight into the location of potential binding sites where OMP-lipid interactions may have a functional importance, such as the *E. coli* outer membrane enzymes OmpT [42]. Other properties of the bilayer in the vicinity of the protein such as bilayer thickness show deviations from bulk properties extending out to 20 Å from the protein surface (see Fig. 4C and Supporting Information Fig. S6). This will be important when we come to explore more crowded membranes in which the separation between adjacent proteins falls within this distance.

### Individual Protein Diffusion

**Figure 5:**
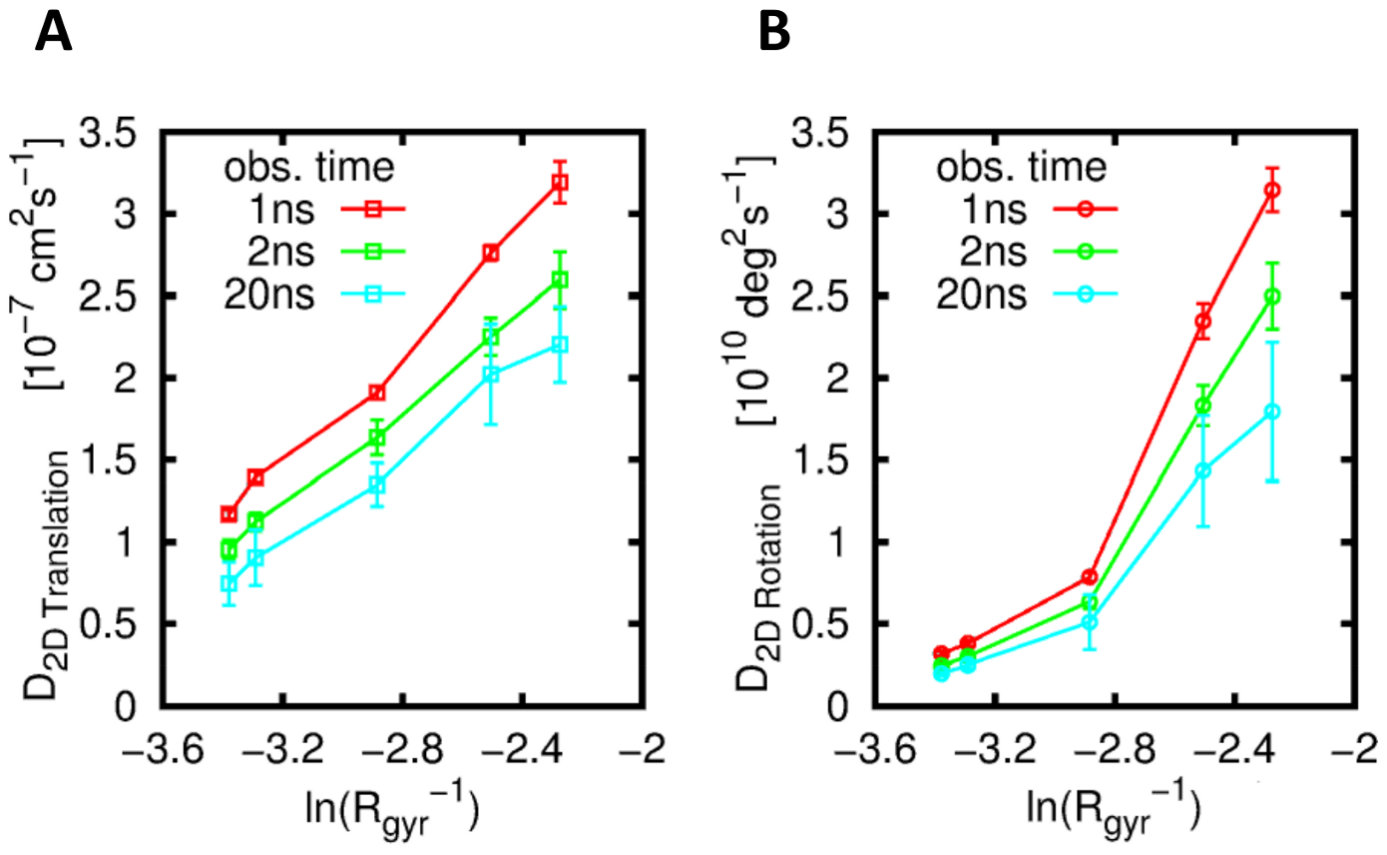
(A) Translational and (B) rotational diffusion of the five OMPs as a function of the logarithm of their inverse radius of gyration (*ln(R_gyr_^−1^)*) for varying observation time (Δt). The proteins are from left to right along the x–axis: LamB, OmpF, FhuA, NanC and OmpA. The standard deviations of the diffusion coefficients calculated from 6×1 µs sections of each 6 µs trajectory are shown as error bars.

The two dimensional rotational and translational diffusion coefficients of all five OMPs were calculated. The translational diffusional coefficients are shown in Fig. 5A as functions of the inverse radius of gyration of the proteins. The OMP:phospholipid number ratio within these simulations was ca. 1∶2500 which ensured two things; firstly that we are calculating diffusion of single proteins in a “bulk” bilayer; and secondly that interactions between periodic images were minimised. The latter is important as a previous MD study [20] has demonstrated the strong influence that system size has on the calculated lipid diffusion). Whilst not a realistic environment bearing in mind the crowded nature of *in vitro* and *in vivo* membranes, these values provide a benchmark against which to compare more complex simulations.

Both the rotational and translational diffusion display a roughly linear correlation with the logarithm of the inverse of the radius of gyration (i.e. *ln(R_gyr_^−1^)*) (see Fig. 5AB and Supporting Information Fig. S7). Although as noted above this correlation is seen in a low protein concentration not representative of an *in vivo* environment, it is to some extent consistent with recent experimental studies of the dependence of membrane protein diffusion rates on protein size [37], [43]–[45], providing an additional degree of confidence in the CG model, although there remains some debate as to the preferred theoretical explanation of these data.

### Multiple Proteins: Lipid & Protein Diffusion in Crowded Bilayers

**Figure 6:**
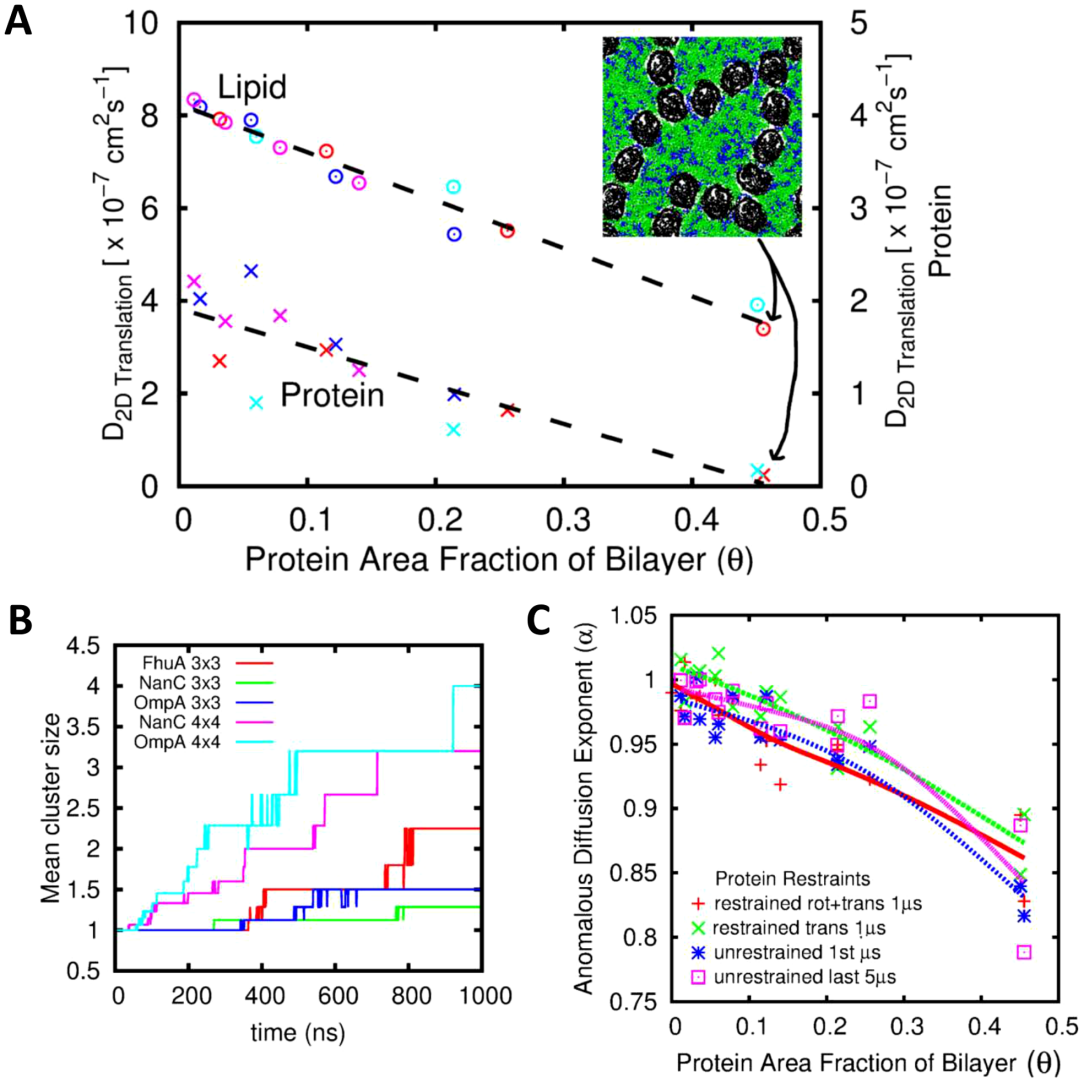
(A) Center of mass diffusion of lipids (circles; left hand axis) and proteins (crosses; right hand axis) as a function of area fraction of bilayer occupied by protein (θ), for Δt = 20 ns. Magenta = OmpA system; dark blue = NanC; red = FhuA; cyan = OmpF. The inset figure shows a snapshot of the 4×4 FhuA system after 1 µs, illustrating how lipids are compartmentalised by a boundary of contiguous interacting proteins. (B) Clustering in the 3×3 and 4×4 simulations, shown using the mean cluster size. (C) Anomalous diffusion exponent α as a function of area fraction of bilayer occupied by protein (θ).

By increasing the number of protein molecules within a membrane patch we are able to explore the effect of increasing the degree of crowding on both lipid and protein diffusion. In Fig. 6A we show the effect of increasing protein concentration on average phospholipid COM diffusion coefficients. Each system was run initially for 1 µs with x–y restraints on all Cα particles, resulting in lipids diffusing amongst a grid of static OMPs. Subsequently, restraints were lifted from all but one central particle per OMP resulting in a further 1 µs simulation of lipids diffusing within a grid of freely rotating OMPs. Before the final 1 µs all restraints were lifted allowing the OMPs to diffuse laterally amongst the lipids. As could be anticipated from results above, the lipid diffusion coefficients are reduced when more proteins are present as more lipids are within 30 Å of a protein. More surprisingly, given the range of individual protein sizes and the dynamics of the systems in terms of clustering, the effect of protein crowding can be captured by collapsing all data points onto a single line fitted to the (translational) diffusion coefficient versus the protein area fraction (θ) of the bilayer (Fig. 6A and Supporting Information Fig. S8). Interestingly, the first two simulations where the proteins were forced to remain on an initial grid showed only a slight reduction in lipid mobility compared to the fully unrestrained system. This suggests that the protein mobility in the unrestrained system is far lower than that of the lipids and can be treated as relatively immobile on the timescale of lipid diffusion. This effect is exaggerated for larger OMPs or clusters of small OMPs. Thus, in a crowded system comparable to an *in vivo* membrane (i.e. θ ca. 0.5) it is clear that lipid will be immobilized by a factor of ca. 2.5 relative to bulk.

There has been considerable discussion recently of anomalous diffusion of lipids in both pure lipid bilayers [24], [25], [46] and in bilayers containing membrane proteins [15], [27]. We believe that the effect of crowding on anomalous diffusion may require a more detailed exploration, using larger simulation systems in order to capture the intricacies involved on longer timescales [15] than is possible in the present study. However, in a preliminary analysis of whether increased protein concentration led to the onset of anomalous diffusion (as suggested by [27]) we calculated α from the MSD of all lipids in each system. This analysis reveals a decrease in the anomalous diffusion exponent α as the protein area fraction θ is increased (Fig. 6C). We note that for the most crowded bilayer (θ>0.4) the anomalous diffusion exponent can be as low as α<0.8. In agreement with recent studies of crowded (θ = 0.34) bilayers containing the NaK channel protein [27] we suggest that the decrease in α is likely a result of restriction of lipid motions due to the proteins surrounding them (see below). In a recent review of experimental and computational studies of membrane diffusion [15] it is noted that whilst there are multiple possible sources of anomalous diffusive dynamics in membranes, the evaluation of the anomalous diffusion parameter α derived from experimental measurements on cellular membranes is challenging. Our simulations suggest that protein crowding within a membrane is a possible source of the observed anomalous diffusion, whilst noting the large difference in timescales between simulation and experimental, and *in vitro* and *in vivo* timescales.

We may also examine the effect on *protein* diffusion of the crowded membranes (Fig. 6A). From these data it is clear that a significant reduction in protein mobility occurs at higher θ values. Thus at θ ca. 0.5 (comparable to cell membranes) translational diffusion coefficients can be reduced to 10% or less of those in bulk membranes (see e.g. points for FhuA and OmpF in Fig. 6A). This provides direct support from the extrapolation from *in vitro* data at lower θ made by e.g. [37] and is consistent with data from FRAP measurements in mammalian cells [47]. Indeed, our simulations and analysis of high θ OmpF-containing membranes are in agreement with the hindered diffusion of OmpF seen in recent high speed AFM of reconstituted OmpF containing membranes [6] although the longer time scale (ca. 1 s) of the latter precludes quantitative comparisons.

We note that during these simulations protein clustering takes place, and so each system may not to be at equilibrium in terms of protein-protein interactions. The relative probability of aggregation occurring within the simulations is controlled by two opposing effects: the size (and hence lateral speed) of each individual protein and the total protein area fraction (θ) of the bilayer. For the 3×3 arrays shown in Fig. 6B, FhuA clusters most rapidly due to a smaller distance needed to travel to encounter another protein whereas NanC and OmpA have to travel further before colliding due to their reduced size. The FhuA 3×3 and NanC 4×4 arrays are roughly equal in terms of θ and here the increased speed of the NanC molecules dominates causing more rapid clustering. For some of the slower systems (2×2 OmpF) clustering is not observed at all over a 1 µs simulation timescale. Thus, the equilibrium state of each system is likely to consist of a large cluster (or network) of proteins surrounded by lipids (cf. [6]). For some of the more crowded systems long two-dimensional ‘chains’ of interacting OMPs are formed that can stretch across periodic boundaries forming one continuous network (Fig. 6A inset)). All lipids are trapped within distinct regions of this network bounded by OMPs, thus severely reducing the long-time diffusion of the lipids through compartmentalisation. We also note a strong orientational preference for ‘tip-to-tip’ interactions, where the interaction surface is limited to a single monomer from each trimer, within our crowded OmpF simulations in contrast to the more varied interactions seen in [6]. However, our simulations have not yet sampled full equilibrium conformations and it is likely that the long-term (ms) evolution of these systems may see rearrangements of the protein-protein interfaces.

## Discussion

We have shown that within our coarse grained lipid simulations the long term diffusive behaviour of bulk lipids is normal with a value of D = 8.5×10^−7^ cm^2^ s^−1^. This compares reasonably well to other reported coarse grained (e.g. [27], [30]) and atomistic (e.g. [24]) simulation values. At short time-scales an anomalous regime exists that is characterised by particle and molecule vibrations. The short term sub-diffusive regime up to 20 ns and the transition to normal Fickian diffusion is also in good agreement with a recent atomistic study of DMPC [24]. Whilst the coarse grained potential captures both of these regimes it also potentially enables us to explore far longer time scales with more lipids and more complex membrane components (i.e. LPS in the bacterial outer membrane or cholesterol in a mammalian membranes). This is essential if we are to attempt to describe the dynamics of even simple *in vitro* systems and eventually *in vivo* bilayers. However, increasing the complexity of the membrane is challenging in terms of computational resources, as the time required for system equilibration may increase substantially, and sampling issues arise for slow moving components. In particular, given observations here and elsewhere [27] of the effects of clustering of interacting proteins in crowded systems on anomalous diffusion of lipids, it will be of considerable interest to extend studies to large crowded systems containing multiple species of lipids and proteins. Such studies will also enable more detailed examination of the effect of clustering on the anomalous diffusion of *proteins* as has been observed in simplified models of membrane protein oligomerization [31]

Experimental studies vary widely in the reported diffusion coefficients of both lipids and proteins depending on the experimental technique used. For example, relatively long time-scale (millisecond) FRAP data predicts lower diffusion coefficients than shorter time-scale (sub-nanosecond) QENS data [48]. Comparison between FRAP data are also complicated by the large variety of the membrane environments studied (GUVs, supported bilayers, in vivo membranes etc.). However, recent FRAP studies of “crowded” GUVs by Ramadurai et al. [37] yielded lipid (DOPC/DOPG: 1.1×10^−7^ cm^2^ s^−1^) and protein (e.g. LacY: 0.4×10^−7^ cm^2^ s^−1^) diffusion coefficients in reasonable agreement with those above. Interestingly, these authors reported some degree of anomalous diffusion (α ca. 0.9) of protein at their higher (3000 µm^−2^) degrees of crowding.

In summary, large scale simulations allow us to probe directly ‘*in vivo*’ regimes which are difficult to address by direct *in vitro* experiments. Thus simulations provide a link between structural level biophysics and studies of complex membranes [6] and cells [49], enabling us to understand emergent spatial and temporal complexities of cell function consequent upon crowding of membrane components.

## Methods

All simulations were run using Gromacs 4.5.3 [50] (www.gromacs.org), and a local modification [51]–[53] of the MARTINI coarse-grained force field [54], [55]. Note that throughout this paper we report simulation times directly without the application of a scaling factor of 4.

The following OMPs (pdb code) were downloaded from www.rcsb.org, stripped of crystallographic waters and other non-protein molecules, and loop regions completed where needed with Modeller 9v8 [56]: FhuA (1BY3), LamB (1AF6), NanC (2WJQ), OmpA (1BXW) and OmpF (2OMF). The atomistic structures were then converted to a coarse-grained structure [54], [55] using the CG protocol described previously [53], with a CG particle representing typically four heavy atoms. Each OMP was energy minimised and embedded into a preformed equilibrated bilayer in 1×1, 2×2, 3×3 and 4×4 grids (where possible). Each membrane patch consists of ca. 75,000 particles with ca. 2500 lipids with overall periodic dimensions of 285×285×105 Å. Two bilayer compositions were used: POPE, and a mixture of POPE:POPG (3∶1) though only the POPE:POPG data is presented in this work. Na^+^ counter ions were added to achieve a neutral electric charge and solvent and lipids equilibrated around the protein for 100 ns with the protein *C*α particles restrained in the x–y plane.

Production runs were then carried out in 3 stages, gradually lifting the restraints on the proteins. For the first 1 µs all Cα particles were restrained (allowing neither rotational or translational diffusion), for the second 1 µs one central particle was restrained in the x–y plane (allowing only rotational diffusion) and for the final 1 µs all restraints were lifted. The reasoning behind this approach was that it would allow an investigation into the effect of embedded objects (OMPs) with varying degrees of mobility on the lipid dynamics. The 1×1 simulations were extended for a further 5 µs (with no restraints) to provide better sampling in the calculation of the diffusion of individual OMPs. All protein positional restraints were of harmonic form with a restoring force of 1000 kJ mol^−1^ nm^−2^ imposed in the x and y directions. We have provided an *mdp* file in the Supporting Information (Supporting Information data file S1) for a typical 1×1 protein in bilayer simulation.

All production simulations are run at 313K µs with Berensden semi–isotropic coupling [53] at 1 bar and separate temperature coupling for the solvent, lipids and protein. A timestep of 20 fs was used, electrostatic interactions are smoothly shifted from zero at 12 Å and Lennard–Jones interaction from 9–12 Å. An elastic network model [57] was used to constrain Cα particles within 7 Å of each other with a force constant of 10 kJ mol^−1^ Å^−2^ to ensure the that the β-barrel structure was preserved.

MDanalysis [58] and in house scripts were used for most trajectory manipulation and analysis. Visualisation was performed in VMD [59] and Pymol [60].

### Calculation of Diffusion Coefficients

#### Lipids

The two dimensional diffusion coefficients of the lipids in all simulations was calculated in two ways.

(1) A fit (at observation time = Δt) to the two–dimensional probability distribution (Eqn. 1) of lipid centre of mass (COM): 
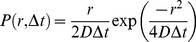
(1)where *P* is the probability density function of *r* after *Δt, D* is the 2-dimensional diffusion coefficient, *Δt* is the observation time, and *r* is the lateral displacement over the period *Δt*. This approach has previously been used by Niemelä et al. [26]. In this method = Δt can be considered a “true” observation time as only particle coordinates at t and t+Δt are used – everything else is discarded. When applied to lipid membrane systems this method often results in different diffusion coefficients at different observation times suggesting diffusion is non linear.

(2) Diffusion is extracted from a plot of mean square displacement versus time by fitting *D_α_* (units cm^2^s^−α^) and *α*. In this study the mean square displacement of the COM of each lipid and the individual head group particles was calculated. For the special case of normal diffusion *α* = 1 and *D* reverts to conventional units of cm^2^s^−1^ (Eqn. 2):
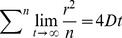
(2)


Sub-diffusion is present when *α<1* (Eqn. 3) as has been observed other studies of lipid membranes:
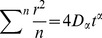
(3)where *D_α_* is the 2-dimensional translational diffusion coefficient, *r* is the lateral displacement at time *t*, and *α* is the anomalous diffusion exponent.

The MSD of sub-trajectories of length *Δt* are averaged over the entire trajectory to improve the convergence. No “restart” time was implemented as large time correlations are present within the system.

#### Proteins

Protein diffusion coefficients are calculated from the mean square displacement (MSD) as a function of time according to the diffusion equations 2 and 4. Δt sub-trajectory values between 1 ns and 200 ns were examined. This method was chosen rather than fitting to a probability distribution due to sampling issues as each simulation only contains one OMP, a single relatively slow moving entity. 6 µs of trajectory was used in the calculation of each OMP diffusion coefficient. To calculate a standard error, diffusion coefficients were calculated for 6×1 µs blocks within each trajectory. The COM of the entire protein was used to calculate translational diffusion. Rotational diffusion was based upon the vector between the COM of two trans-membrane halves of the protein split equally down the middle: 
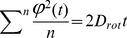
(4)where *D_rot_* is the 2-dimensional rotational diffusion coefficient, and *φ(t)* is the rotation at time *t*.

## Supporting Information

Figure S1 **Mean Square Displacement versus time for lipid center of mass (COM) and head-group particles.** The inset is the same data on a non-log scale. The data is from a circa 2500 lipid only POPE:POPG system run for 6 µs and the MSD is averaged over intervals of 500 ns. Anomalous diffusion is seen at t<30 ns.
(PDF)

Figure S2 **2D-Lipid displacement distributions at varying observation times for a circa 2500 lipid only POPE:POPG system run for 6 µs.** The diffusion coefficient is extracted from the fit to the probability density function.
(PDF)

Figure S3 **Phospholipid diffusion coefficients for the outer leaflet of the bilayer as a function of distance from protein (NanC – top panels, OmpF - bottom panels) and of observation time.** Diffusion is calculated by tracking three different phospholipid particles; PO4^−^ (left panels), NH3^+^ (middle panels) and GLH (right panels). Each point represents the diffusion of lipids within annuli of 10 Å width (i.e. a point at 5 Å represents lipids within the first annulus 0–10 Å from the protein surface). The data on each plot are calculated from 6 µ*s* trajectories of a single protein in a 3∶1 POPE:POGE bilayer.
(PDF)

Figure S4 Top panels: Leaflet asymmetry of diffusion coefficients illustrated for all proteins. Ratio of inner to outer leaflet COM diffusion coefficients as a function of distance from protein and of observation time. Middle panels: Coarse-grained models of the corresponding proteins coloured on time averaged number of protein contacts (cutoff 7 Å) to lipid phosphate particles on a blue (0%) to red (100%) scale. Bottom panels: Coarse grained models of the corresponding proteins displaying the location of charged residues for each protein, red (acidic) and blue (basic).
(PDF)

Figure S5 **Time averaged two–dimensional phosphate particle densities around each protein for the outer (top panels) and inner (bottom panels) leaflets.** Proximal acidic/basic residues are shown as blue/red points. The Cα trace is shown in black.
(PDF)

Figure S6 **Time averaged two–dimensional bilayer distortions from bulk thickness in the vicinity of each protein.** Bilayer thickness is calculated based on the minimum distance between the two closest PO4 particles in opposing leaflets. Acidic/basic residues are shown as blue/red points. The Cα trace is shown in black.
(PDF)

Figure S7 **Translational (A) and Rotational (B) diffusion of the five OMPs as a function of the logarithm of their inverse radius of gyration (** ***ln(R_gyr_^−1^)*** **) for varying observation time (** ***Δt*** **).** The proteins are from left to right along the x–axis: LamB, OmpF, FhuA, NanC and OmpA. The standard deviations of the diffusion coefficients calculated from 6×1 µs sections of each 6 µs trajectory are shown as error bars.
(PDF)

Figure S8 **Center of mass diffusion of phospholipids as a function of area fraction of bilayer occupied by protein (θ).** Observation time *Δt* = 20 ns. Magenta = OmpA system; dark blue = NanC; red = FhuA; cyan = OmpF. The left panel is the system with freely diffusing proteins; the central panel relates to a grid of OMPs with a central particle restrained in the x–y plane (rotation but not translation allowed in the bilayer plane); the right panel is a grid of OMPs with all Cα particles restrained in the x–y plane (neither rotation or translation allowed in the bilayer plane).
(PDF)

Table S1 **A summary of some of the major properties of the five OMP species involved in our study.** Number of residues, *R* ***_gyr_*** and net charge refer to the structures used in this study in their stated oligomeric state. Charge asymmetry (Outer/Inner) is defined from the 1×1 embedded protein simulations as the number of charged residues that pass within 5 Å of a lipid head group particle in the respective leaflets.
(PDF)

Data File S1 **An example of an** ***mdp*** **file for a typical 1×1 protein in bilayer simulation.**
(PDF)
