# Supplementary figures and images for: Reduced Lateral Mobility of Lipids and Proteins in Crowded Membranes
Source: PLoS Comput Biol. 2013 Apr 11;9(4):e1003033. doi: 10.1371/journal.pcbi.1003033 (PMC3623704; doi:10.1371/journal.pcbi.1003033)

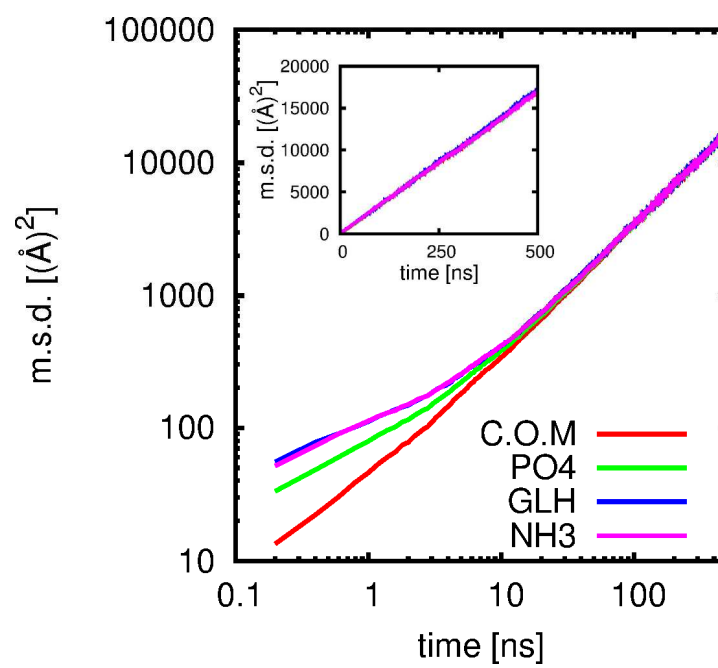

Supplement: Figure S1 — Mean Square Displacement versus time for lipid center of mass (COM) and head-group particles. The inset is the same data on a non-log scale. The data is from a circa 2500 lipid only POPE:POPG system run for 6 µs and the MSD is averaged over intervals of 500 ns. Anomalous diffusion is seen at t<30 ns. (PDF) [file pcbi.1003033.s001.pdf]

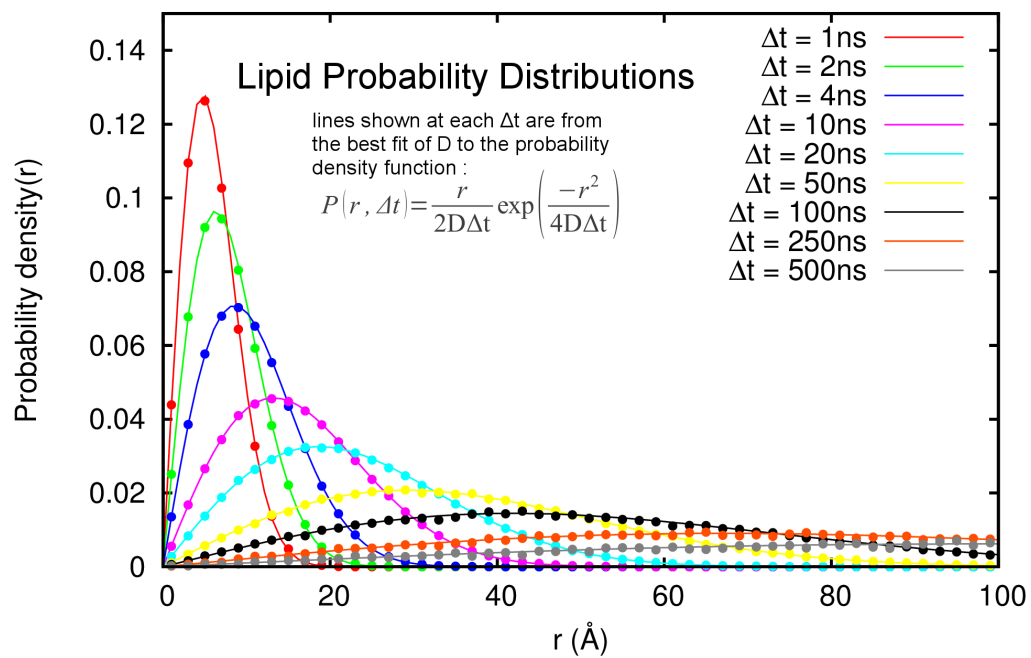

Supplement: Figure S2 — 2D-Lipid displacement distributions at varying observation times for a circa 2500 lipid only POPE:POPG system run for 6 µs. The diffusion coefficient is extracted from the fit to the probability density function. (PDF) [file pcbi.1003033.s002.pdf]

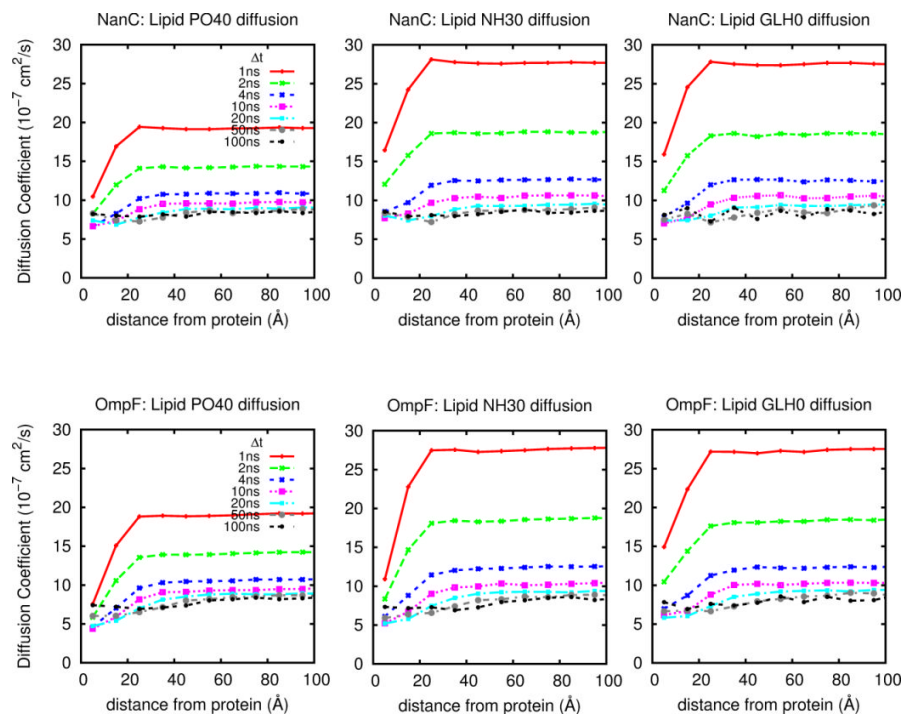

Supplement: Figure S3 — Phospholipid diffusion coefficients for the outer leaflet of the bilayer as a function of distance from protein (NanC – top panels, OmpF - bottom panels) and of observation time. Diffusion is calculated by tracking three different phospholipid particles; PO4− (left panels), NH3+ (middle panels) and GLH (right panels). Each point represents the diffusion of lipids within annuli of 10 Å width (i.e. a point at 5 Å represents lipids within the first annulus 0–10 Å from the protein surface). The data on each plot are calculated from 6 µs trajectories of a single protein in a 3∶1 POPE:POGE bilayer. (PDF) [file pcbi.1003033.s003.pdf]

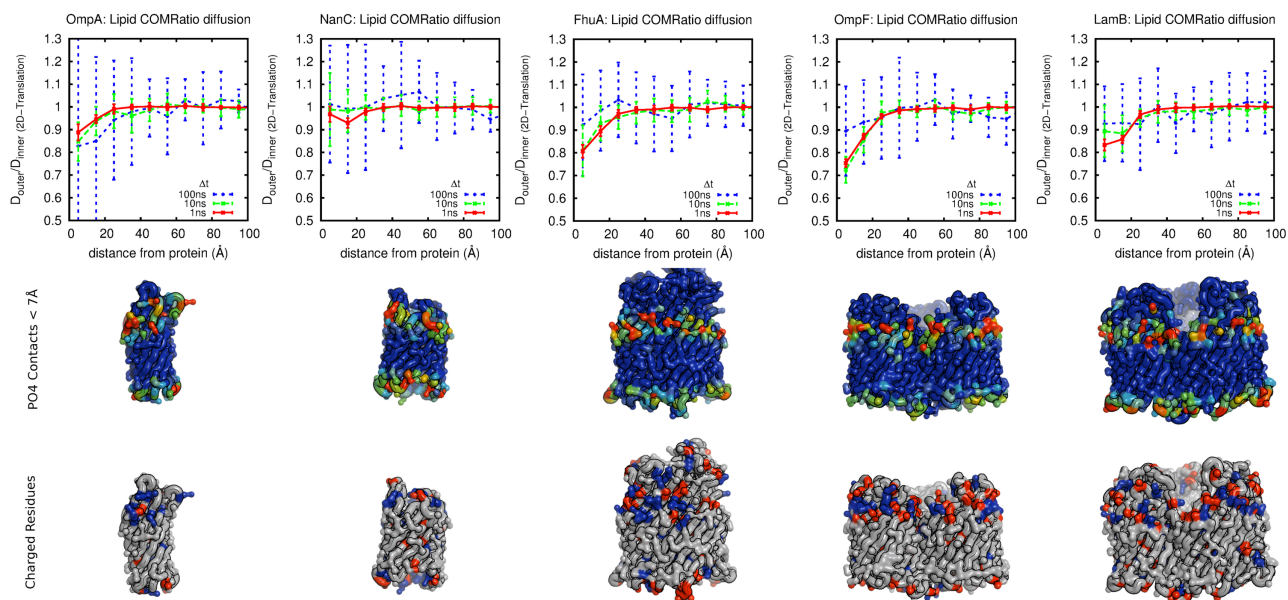

Supplement: Figure S4 — Top panels: Leaflet asymmetry of diffusion coefficients illustrated for all proteins. Ratio of inner to outer leaflet COM diffusion coefficients as a function of distance from protein and of observation time. Middle panels: Coarse-grained models of the corresponding proteins coloured on time averaged number of protein contacts (cutoff 7 Å) to lipid phosphate particles on a blue (0%) to red (100%) scale. Bottom panels: Coarse grained models of the corresponding proteins displaying the location of charged residues for each protein, red (acidic) and blue (basic). (PDF) [file pcbi.1003033.s004.pdf]

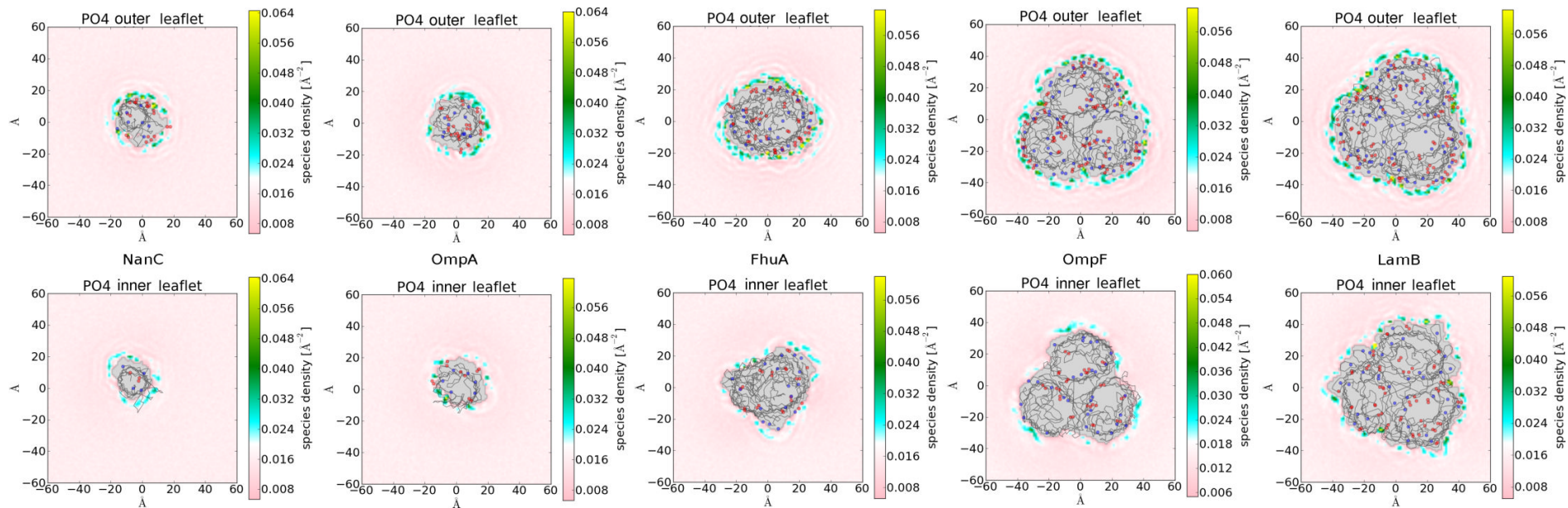

Supplement: Figure S5 — Time averaged two–dimensional phosphate particle densities around each protein for the outer (top panels) and inner (bottom panels) leaflets. Proximal acidic/basic residues are shown as blue/red points. The Cα trace is shown in black. (PDF) [file pcbi.1003033.s005.pdf]

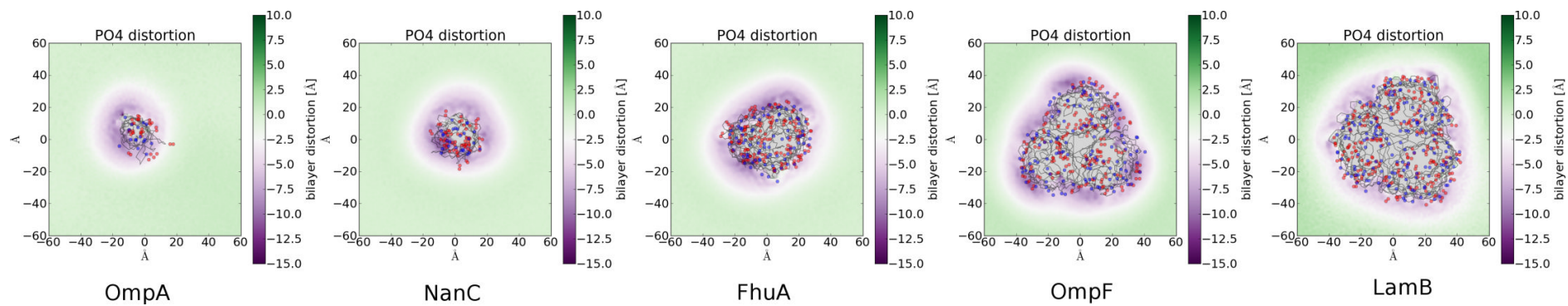

Supplement: Figure S6 — Time averaged two–dimensional bilayer distortions from bulk thickness in the vicinity of each protein. Bilayer thickness is calculated based on the minimum distance between the two closest PO4 particles in opposing leaflets. Acidic/basic residues are shown as blue/red points. The Cα trace is shown in black. (PDF) [file pcbi.1003033.s006.pdf]

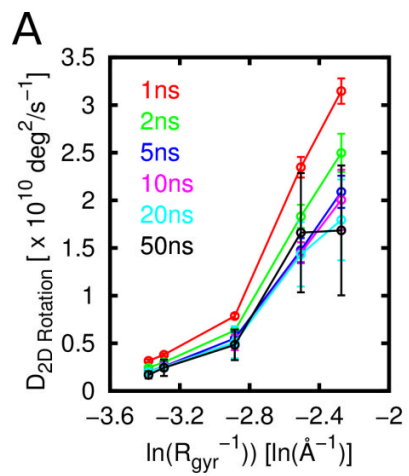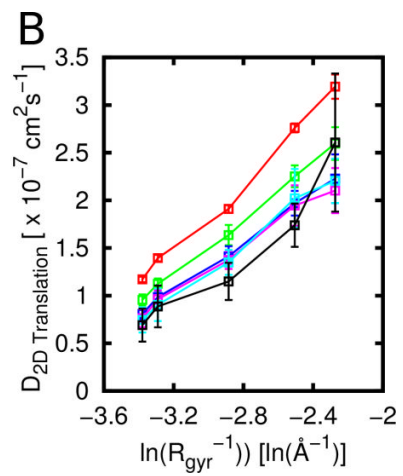

Supplement: Figure S7 — Translational (A) and Rotational (B) diffusion of the five OMPs as a function of the logarithm of their inverse radius of gyration ( ln(Rgyr−1) ) for varying observation time ( Δt ). The proteins are from left to right along the x–axis: LamB, OmpF, FhuA, NanC and OmpA. The standard deviations of the diffusion coefficients calculated from 6×1 µs sections of each 6 µs trajectory are shown as error bars. (PDF) [file pcbi.1003033.s007.pdf]

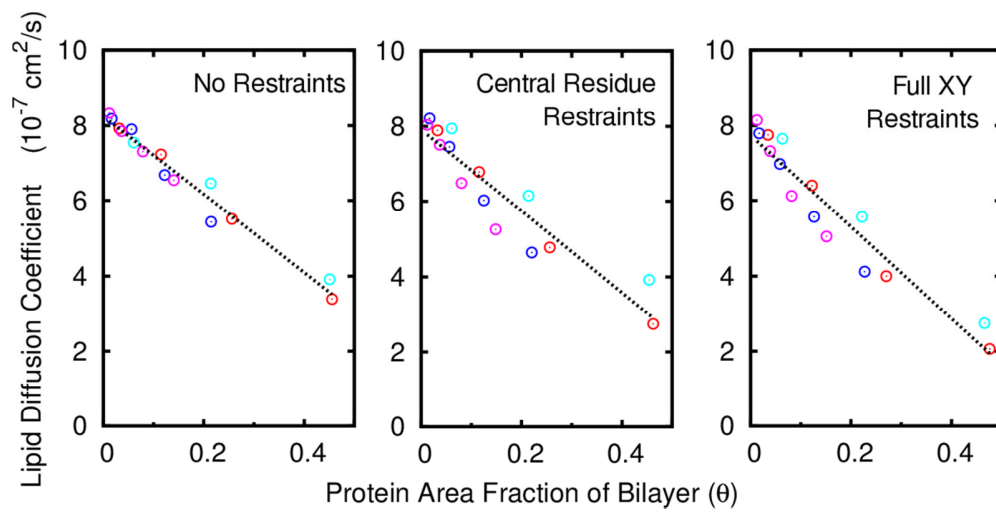

Supplement: Figure S8 — Center of mass diffusion of phospholipids as a function of area fraction of bilayer occupied by protein (θ). Observation time Δt = 20 ns. Magenta = OmpA system; dark blue = NanC; red = FhuA; cyan = OmpF. The left panel is the system with freely diffusing proteins; the central panel relates to a grid of OMPs with a central particle restrained in the x–y plane (rotation but not translation allowed in the bilayer plane); the right panel is a grid of OMPs with all Cα particles restrained in the x–y plane (neither rotation or translation allowed in the bilayer plane). (PDF) [file pcbi.1003033.s008.pdf]
