# Supplementary material for: Reduced Lateral Mobility of Lipids and Proteins in Crowded Membranes
Source: PLoS Comput Biol. 2013 Apr 11;9(4):e1003033. doi: 10.1371/journal.pcbi.1003033 (PMC3623704; doi:10.1371/journal.pcbi.1003033)
Supplement: Table S1 — A summary of some of the major properties of the five OMP species involved in our study. Number of residues, R gyr and net charge refer to the structures used in this study in their stated oligomeric state. Charge asymmetry (Outer/Inner) is defined from the 1×1 embedded protein simulations as the number of charged residues that pass within 5 Å of a lipid head group particle in the respective leaflets. (PDF) [file pcbi.1003033.s009.pdf]

Table S1

A summary of some of the major properties of the five OMP species involved in our study. Number of residues, R_{gyr} and net charge refer to the structures used in this study in their stated oligomeric state. Charge asymmetry (Outer/Inner) is defined from the 1x1 embedded protein simulations as the number of charged residues that pass within 5 Å of a lipid head group particle in the respective leaflets.

OMP	No. of residues	R_{gyr} (Å)	Oligomerisation state	Net charge	Notes	Charge asymmetry (outer/inner)
**FhuA**	695	18.0	monomer	-12	Transporter (ferrichrome)	46/26
**LamB**	1263	29.3	trimer	-57	Porin (oligosaccharides)	72/36
**NanC**	215	12.3	monomer	-1	Porin (N-acetylneuraminic acid)	21/13
**OmpA**	171	9.7	monomer	-3	Possibly structural. Periplasmic domain not included.	18/5
**OmpF**	1020	26.8	trimer	-36	Porin (cation selective)	72/18
