# Supplementary material for: Reduced Lateral Mobility of Lipids and Proteins in Crowded Membranes
Source: PLoS Comput Biol. 2013 Apr 11;9(4):e1003033. doi: 10.1371/journal.pcbi.1003033 (PMC3623704; doi:10.1371/journal.pcbi.1003033)
Supplement: Data File S1 — An example of an mdp file for a typical 1×1 protein in bilayer simulation. (PDF) [file pcbi.1003033.s010.pdf]

**Supporting Information Data File S1:**

This provides an example of an *mdp* file for for a typical 1x1 protein in bilayer simulation.

```
; VARIOUS PREPROCESSING OPTIONS
; Preprocessor information: use cpp syntax.
; e.g.: -I/home/joe/doe -I/home/mary/hoe
include                  =
; e.g.: -DI_Want_Cookies -DMe_Too
define                   =

; RUN CONTROL PARAMETERS
integrator               = md
; Start time and timestep in ps
tinit                    = 0.0
dt                       = 0.02
nsteps                   = 50000000
; For exact run continuation or redoing part of a run
; Part index is updated automatically on checkpointing (keeps files
separate)
simulation_part          = 1
init_step                = 0
; mode for center of mass motion removal
comm-mode                = Linear
; number of steps for center of mass motion removal
nstcomm                  = 1
; group(s) for center of mass motion removal
comm-grps                = PROTEIN+LIPID

; LANGEVIN DYNAMICS OPTIONS
; Friction coefficient (amu/ps) and random seed
bd-fric                  = 0
ld-seed                  = 1993

; ENERGY MINIMIZATION OPTIONS
; Force tolerance and initial step-size
emtol                    = 10
emstep                   = 0.01
; Max number of iterations in relax_shells
niter                    = 20
; Step size (ps^2) for minimization of flexible constraints
fcstep                   = 0
; Frequency of steepest descents steps when doing CG
nstcgsteep               = 1000
nbfgscorr                = 10

; TEST PARTICLE INSERTION OPTIONS
rtpi                     = 0.05

; OUTPUT CONTROL OPTIONS
; Output frequency for coords (x), velocities (v) and forces (f)
nstxout                  = 5000000
```

```
nstvout                 = 5000000
nstfout                 = 5000000
; Output frequency for energies to log file and energy file
nstlog                  = 1000000
nstenergy               = 1000000
; Output frequency and precision for xtc file
nstxtcout               = 10000
xtc_precision           = 1000
; This selects the subset of atoms for the xtc file. You can
; select multiple groups. By default all atoms will be written.
xtc-grps                = PROTEIN LIPID
; Selection of energy groups
energygrps              = PROTEIN LIPID SOLVENT

; NEIGHBORSEARCHING PARAMETERS
; nblist update frequency
nstlist                 = 10
; ns algorithm (simple or grid)
ns-type                 = Grid
; Periodic boundary conditions: xyz, no, xy
pbc                     = xyz
periodic_molecules      = no
; nblist cut-off
rlist                   = 1.3

; OPTIONS FOR ELECTROSTATICS AND VDW
; Method for doing electrostatics
coulombtype             = Shift
rcoulomb_switch         = 0.0
rcoulomb                = 1.2
; Relative dielectric constant for the medium and the reaction field
epsilon_r               = 20
epsilon_rf              = 1
; Method for doing Van der Waals
vdw_type                = Shift
; cut-off lengths
rvdw_switch             = 0.9
rvdw                    = 1.2
; Apply long range dispersion corrections for Energy and Pressure
DispCorr                = No
; Extension of the potential lookup tables beyond the cut-off
table-extension         = 1
; Seperate tables between energy group pairs
energygrp_table         =
; Spacing for the PME/PPPM FFT grid
fourierspacing          = 0.12
; FFT grid size, when a value is 0 fourierspacing will be used
fourier_nx              = 0
fourier_ny              = 0
fourier_nz              = 0
; EWALD/PME/PPPM parameters
pme_order               = 4
ewald_rtol              = 1e-05
```

```
ewald_geometry           = 3d
epsilon_surface          = 0
optimize_fft             = no

; IMPLICIT SOLVENT ALGORITHM
implicit_solvent         = No

; GENERALIZED BORN ELECTROSTATICS
; Algorithm for calculating Born radii
gb_algorithm             = Still
; Frequency of calculating the Born radii inside rlist
nstgbradii               = 1
; Cutoff for Born radii calculation; the contribution from atoms
; between rlist and rgbradii is updated every nstlist steps
rgbradii                 = 1
; Dielectric coefficient of the implicit solvent
gb_epsilon_solvent       = 80
; Salt concentration in M for Generalized Born models
gb_saltconc              = 0
; Scaling factors used in the OBC GB model. Default values are OBC(II)
gb_obc_alpha             = 1
gb_obc_beta              = 0.8
gb_obc_gamma             = 4.85
; Surface tension (kJ/mol/nm^2) for the SA (nonpolar surface) part of
GBSA
; The default value (2.092) corresponds to 0.005 kcal/mol/Angstrom^2.
sa_surface_tension       = 2.092

; OPTIONS FOR WEAK COUPLING ALGORITHMS
; Temperature coupling
tcoupl                   = Berendsen
; Groups to couple separately
tc-grps                  = PROTEIN LIPID SOLVENT
; Time constant (ps) and reference temperature (K)
tau_t                    = 4.0 4.0 4.0
ref_t                    = 313 313 313
; Pressure coupling
Pcoupl                   = Berendsen
Pcoupltype               = semiisotropic
; Time constant (ps), compressibility (1/bar) and reference P (bar)
tau_p                    = 4   4  4
compressibility          = 5e-6 5e-6  5e-6
ref_p                    = 1.0 1.0  1.0
; Scaling of reference coordinates, No, All or COM
refcoord_scaling         = No
; Random seed for Andersen thermostat
andersen_seed            = 815131

; OPTIONS FOR QMMM calculations
QMMM                     = no
; Groups treated Quantum Mechanically
QMMM-grps                =
; QM method
```

```
QMmethod                 =
; QMMM scheme
QMMMscheme               = normal
; QM basisset
QMbasis                  =
; QM charge
QMcharge                 =
; QM multiplicity
QMmult                   =
; Surface Hopping
SH                       =
; CAS space options
CASorbitals              =
CASelectrons             =
SAon                     =
SAoff                    =
SAsteps                  =
; Scale factor for MM charges
MMChargeScaleFactor      = 1
; Optimization of QM subsystem
bOPT                     =
bTS                      =

; SIMULATED ANNEALING
; Type of annealing for each temperature group (no/single/periodic)
annealing                =
; Number of time points to use for specifying annealing in each group
annealing_npoints        =
; List of times at the annealing points for each group
annealing_time           =
; Temp. at each annealing point, for each group.
annealing_temp           =

; GENERATE VELOCITIES FOR STARTUP RUN
gen-vel                  = no
gen_temp                 = 313
gen_seed                 = 1

; OPTIONS FOR BONDS
constraints              = none
; Type of constraint algorithm
constraint_algorithm     = Lincs
; Do not constrain the start configuration
continuation             = no
; Use successive overrelaxation to reduce the number of shake
iterations
Shake-SOR                = no
; Relative tolerance of shake
shake-tol                = 0.0001
; Highest order in the expansion of the constraint coupling matrix
lincs-order              = 4
; Number of iterations in the final step of LINCS. 1 is fine for
; normal simulations, but use 2 to conserve energy in NVE runs.
```

```
; For energy minimization with constraints it should be 4 to 8.
lincs-iter               = 1
; Lincs will write a warning to the stderr if in one step a bond
; rotates over more degrees than
lincs-warnangle          = 30
; Convert harmonic bonds to morse potentials
morse                    = no

; ENERGY GROUP EXCLUSIONS
; Pairs of energy groups for which all non-bonded interactions are
excluded
energygrp_excl           =

; WALLS
; Number of walls, type, atom types, densities and box-z scale factor
for Ewald
nwall                    = 0
wall_type                = 9-3
wall_r_linpot            = -1
wall_atomtype            =
wall_density             =
wall_ewald_zfac          = 3

; COM PULLING
; Pull type: no, umbrella, constraint or constant_force
pull                     = no

; NMR refinement stuff
; Distance restraints type: No, Simple or Ensemble
disre                    = simple
; Force weighting of pairs in one distance restraint: Conservative or
Equal
disre_weighting          = Equal
; Use sqrt of the time averaged times the instantaneous violation
disre_mixed              = no
disre_fc                 = 1000
disre_tau                = 50
; Output frequency for pair distances to energy file
nstdisreout              = 100
; Orientation restraints: No or Yes
orire                    = no
; Orientation restraints force constant and tau for time averaging
orire-fc                 = 0
orire-tau                = 0
orire-fitgrp             =
; Output frequency for trace(SD) and S to energy file
nstorireout              = 100
; Dihedral angle restraints: No or Yes
dihre                    = no
dihre-fc                 = 1000

; Free energy control stuff
free-energy              = no
```

```
init-lambda              = 0
delta-lambda             = 0
sc-alpha                 = 0
sc-power                 = 0
sc-sigma                 = 0.3
couple-moltype           =
couple-lambda0           = vdw-q
couple-lambda1           = vdw-q
couple-intramol          = no

; Non-equilibrium MD stuff
acc-grps                 =
accelerate               =
freezegrps               =
freezedim                =
cos-acceleration         = 0
deform                   =

; Electric fields
; Format is number of terms (int) and for all terms an amplitude (real)
; and a phase angle (real)
E-x                      =
E-xt                     =
E-y                      =
E-yt                     =
E-z                      =
E-zt                     =

; User defined thingies
user1-grps               =
user2-grps               =
userint1                 = 0
userint2                 = 0
userint3                 = 0
userint4                 = 0
userreal1                = 0
userreal2                = 0
userreal3                = 0
userreal4                = 0
```
